# Eastern South African hydroclimate over the past 270,000 years

**DOI:** 10.1038/srep18153

**Published:** 2015-12-21

**Authors:** Margit H. Simon, Martin Ziegler, Joyce Bosmans, Stephen Barker, Chris J.C. Reason, Ian R. Hall

**Affiliations:** 1 School of Earth and Ocean Sciences, Cardiff University, Cardiff, CF10 3AT, United Kingdom; 2 Faculty of Geosciences, Utrecht University, 3584 CD Utrecht, Netherlands; 3 Department of Oceanography, University of Cape Town, South Africa

## Abstract

Processes that control the hydrological balance in eastern South Africa on orbital to millennial timescales remain poorly understood because proxy records documenting its variability at high resolution are scarce. In this work, we present a detailed 270,000 year-long record of terrestrial climate variability in the KwaZulu-Natal province based on elemental ratios of Fe/K from the southwest Indian Ocean, derived from X-ray fluorescence core scanning. Eastern South African climate variability on these time scales reflects both the long-term effect of regional insolation changes driven by orbital precession and the effects associated with high-latitude abrupt climate forcing over the past two glacial-interglacial cycles, including millennial-scale events not previously identified. Rapid changes towards more humid conditions in eastern South Africa as the Northern Hemisphere entered phases of extreme cooling were potentially driven by a combination of warming in the Agulhas Current and shifts of the subtropical anticyclones. These climate oscillations appear coherent with other Southern Hemisphere records but are anti-phased with respect to the East Asian Monsoon. Numerical modelling results reveal that higher precipitation in the KwaZulu-Natal province during precession maxima is driven by a combination of increased local evaporation and elevated moisture transport into eastern South Africa from the coast of Mozambique.

The latitudinal extent of Africa is divided by the equator into two nearly equal parts, resulting in distinct rainfall zones in each hemisphere (Fig. 1). The African hydroclimate is complex and sensitive to both regional oceanic and remote forcing such as ENSO^1^. Studies documenting its climate variability are adding towards the growing number of high-resolution and absolutely dated palaeoclimate records from both the Northern and Southern Hemispheres, thereby contributing to the understanding of global rainfall patterns on orbital- and millennial timescales (Fig. 1). Nevertheless, the full global scale documentation suffers from gaps in regional rainfall records in both Hemispheres.

During the late Pleistocene, changes in African climate were partly paced by periodic variations in insolation resulting from Earth’s orbital precession (23–19 ka)^2^. Orbital precession leads to anti-phased summer insolation maxima between the hemispheres, resulting in an interhemispheric temperature contrast and latitudinal shifts of the Inter Tropical Convergence Zone (ITCZ) and associated regions that are affected by summer monsoonal precipitation^3^. Moreover African climate was sensitive to high-latitude forcing predominately varying on glacial-interglacial timescales related to orbital obliquity and eccentricity changes (41 and 100 ka cycles)^2^. Although in many areas of the African tropics the climate is considered to have been strongly influenced by the movement of the ITCZ and associated rainbelts^2^, studies from the central and eastern African tropics show that, additionally, a mixture of Atlantic and Indian Ocean related mechanisms account for reconstructed trends in the moisture balance^4^,^5^,^6^ (Fig. 1). The importance of other convergence zones such as the Congo Air Boundary has been highlighted in controlling the influx of Atlantic moisture to the East African region during the last glacial cycle^7^. Simulations using the Community Climate System Model version 3 (CCSM3) suggest that changes in greenhouse gas (GHG) concentrations, rather than local insolation forcing (or the combination of both), ultimately determine the hydrological balance of the African continent during the last deglaciation^8^, especially in southeastern equatorial Africa.

The palaeohydrology of the African continent is documented by a variety of terrestrial (lake) and offshore marine records with the history of the north- and southeastern tropical African climate, e.g.^4^,^5^,^6^,^7^,^9^,^10^,^11^,^12^, being better resolved than its southernmost counterpart, e.g.^13^,^14^,^15^,^16^ (Fig. 1). In that region, long-term climate shifts have been related to fluctuations in the positioning of the ITCZ, the Antarctic polar vortex and westerly storm tracks^17^. However, to build a more coherent picture of the evolution of African rainfall variability, long high-resolution records documenting subtropical South African climate variability are essential but currently lacking. Future climate change projections from the latest phase of the Coupled Model Intercomparison Project suggest that severe droughts will impact South Africa by the end of the 21^st^ Century, due to changes in the Hadley Circulation and increased surface temperatures, with potentially significant socio-economic consequences for that region^18^. Palaeoclimate reconstructions provide evidence of past long-term and abrupt climate variability in South Africa and can provide an important test-bed for validating such model predictions. Moreover, such records are important archives of past climate changes which, when considered in combination with archaeological records, can provide evidence for the potential link between climate and human settlement in South Africa during the Middle Stone Age^19^. Here we present a geochemical record that provides a detailed climate history for eastern South Africa over the past 270,000 years, thereby filling an important gap in existing records.

## Environmental setting

**Figure 1:**
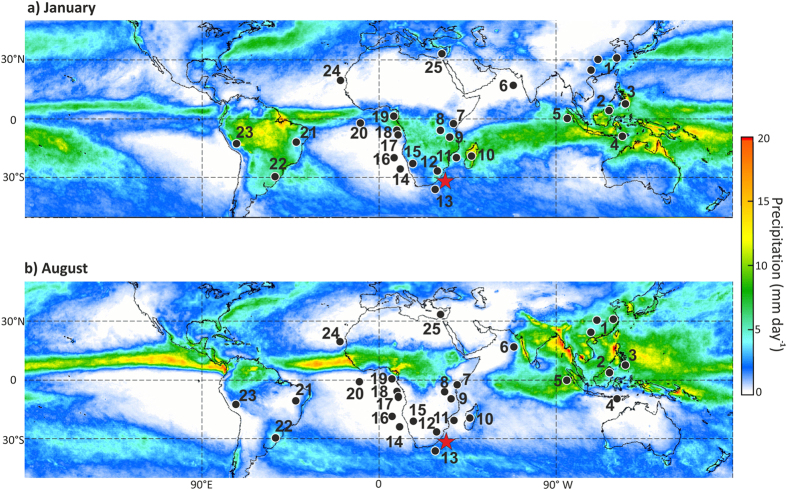
Global modern precipitation rates estimated from satellite imagery of the Tropical Rainfall Measuring Mission (**a**) January (**b**) August. Map was taken from NASA homepage http://trmm.gsfc.nasa.gov/ and modified thereafter. Colours indicate modern rainfall rates recorded in mm per month. The climate over large parts of Africa is characterised by a strong seasonality with summer rainfall and the approximate position of the ITCZ and its associated rainbelt migrating between the North and South continent over the course of the year. Sites mentioned in the main text: CD154 10-06P (this study, red star), southwest Indian Ocean and reference sites (black dots). 1-Hulu, Dongge and Sanbao Caves^40^,^41^,^44^, 2- Gunung Buda/Gunung Mulu Caves, Borneo^39^, 3- Core MD06–3075 Davao Gulf on the southern side of Mindanao, Philippines^34^ 4- Liang Luar Cave, Indonesia^36^, 5- Cores SO189-119KL; SO189–144KL, SO189-39KL, western coast of Sumatra^54^, 6- SO130-289KL in the northeastern Arabian Sea^55^, 7-Cores of Lake Challa^7^, 8- Cores NP04-KH04-3A-1K and NP04-KH04-4A-1K, Lake Tanganyika^6^, 9- Core M98–1P and M98–2PG, north basin of Lake Malawi^4^,^10^, 10- Lake Tritrivakely, Madagascar^15^, 11- Core GeoB9307-3 Zambezi river mouth^33^, 12-Lake Tswaing in southeastern Africa^16^, 13- Core CD154 17-17K, southwest Indian Ocean^19^, 14- Core MD08-3167, South Atlantic^14^, 15-Spitzkoppe hyrax midden, southwest Africa^42^, 16-Core MD96-2094,Walvis Ridge^17^, 17- Core GeoB 6518-1, Congo River^5^, 18- Core GeoB1008-3, Zaire River delta^12^, 19- Core MD03-2707, Gulf of Guinea^9^, 20- ODP Site 663 eastern equatorial Atlantic^2^, 21- Lapa dos Brejo˜es and Toca da Barriguda caves, north eastern Brazil^58^, 22- Caverna Botuvera, southern Brazil^35^,^38^, 23-Pacupahuain Cave in the central Peruvian Andes^57^, 24-Core GeoB7920, northwest Africa^56^, 25-Eastern Mediterranean (ODP Site 968)^11^.

Subtropical South Africa is located in the transition between tropical and mid-latitudinal climate systems, as well as being near the Indian, Atlantic and Southern Oceans. The largest part of southern Africa receives most of its rainfall in austral summer (December-February). The winter rainfall zone in southwestern South Africa is influenced by mid-latitude cold fronts and cut-off lows with rainfall amounts strongly influenced by orography and distance from the coast. Located on the east coast of subtropical South Africa, KwaZulu-Natal (KZN) (Fig. 1) receives mainly summer rainfall due to ridging anticyclones, cut-off lows, mesoscale convective complexes and tropical- to extratropical cloud bands^20^,^21^ which are influenced by conditions in the South Indian Ocean as well as by ENSO. Anticyclonic ridging events produce onshore flow which, when strong and moist enough, can lead to orographic rainfall due to the hilly coastline of southeast Africa^20^ and mountainous interior. Cut-off low pressure systems are cold-cored depressions in mid-latitudes which are often linked with deep and moist convection and heavy rainfall^21^. Tropical to extratropical cloud bands form in summer as cold fronts passing south of the landmass, link up with a disturbance over tropical southern Africa, as a result of which substantial rainfall may occur over KZN and elsewhere^21^.

To investigate eastern South African climate variability over the last two glacial-interglacial cycles we analysed marine sediment core CD154-10-06P (31°10.36′ S, 032° 08.91′ E, 3076 m water depth) retrieved from the southwest Indian Ocean ~160 km off the KZN coast during *RRS Charles Darwin* cruise number 154 (Fig. 1). Several rivers traverse KZN before flowing into the adjacent southwest Indian Ocean. Most of these rivers carry high sediment loads and are determined by their typical brown-waters^22^. High precipitation promotes intense chemical weathering of bedrock^23^ in tropical humid regions such as KZN, resulting in highly weathered, iron-rich soils which are transported to the sea by rivers^24^. The closest source of terrestrial input to our core site is the Thukela River situated north of Durban, which is some 502 km long, has a catchment of ~29,000 km^2^ and an annual mean freshwater discharge of 3,865 × 10^6^ m^3^ (ref. 25). Elemental concentrations along the entire core were obtained with an X-ray fluorescence (XRF) core scanner (ITRAX), (Methods). As a first order approximation, bulk element measurements on selected samples (n = 18) in the CD154 10–06 P record show that the average Fe/K ratios (ln(Fe/K) = 1.60 ± 0.23) at our core location are similar to those of the suspended sediment load of South African rivers draining similar rock types as the Thukela and Great Kei rivers, (ln(Fe/K = Ø 1.16)^24^. A study on ^87^Sr/^86^Sr ratios from core top and Last Glacial Maximum samples at/or close to our core location provided evidence that the sediment delivered to the sites by the river system is a mixture of the various local southern African source terrains^26^. Moreover, we analysed a set of 39 samples for the branched isoprenoid tetraether (BIT) index which is a proxy for the relative abundance of terrestrial organic matter^27^. Overall, elevated BIT index ratios correspond to intervals of higher Fe/K ratios thus suggesting that the terrestrial material delivered to the CD154 10-06P core site is of local terrestrial origin (Supplementary Figure (SF) 3, Supplementary Information (SI)). High Fe/K ratios in marine sediments have been interpreted to indicate high soil erosion and/or enhanced chemical weathering processes on land due to a humid climate^28^,^29^. Both processes are a result of increased precipitation, which generates highly weathered soils typical for tropical and subtropical environments. As clay minerals are intensely weathered, the structure of silicate clays changes as they lose silica, the remaining soil becoming enriched in iron oxides, which is ultimately recorded as high Fe/K values in the sediment (SI). Likewise, changes between low and high Fe/K ratios can be a result of runoff variability and associated soil erosion without any prior changes in bedrock weathering. We therefore concede that a distinction between these processes is crucial and remains a challenging task. Nevertheless, changes in runoff would still suggest a shift in the climatic regime on land between intervals of more frequent and/or intense rainfall and vice versa.

## Discussion

### Long-term variability in eastern South African hydroclimate

**Figure 2:**
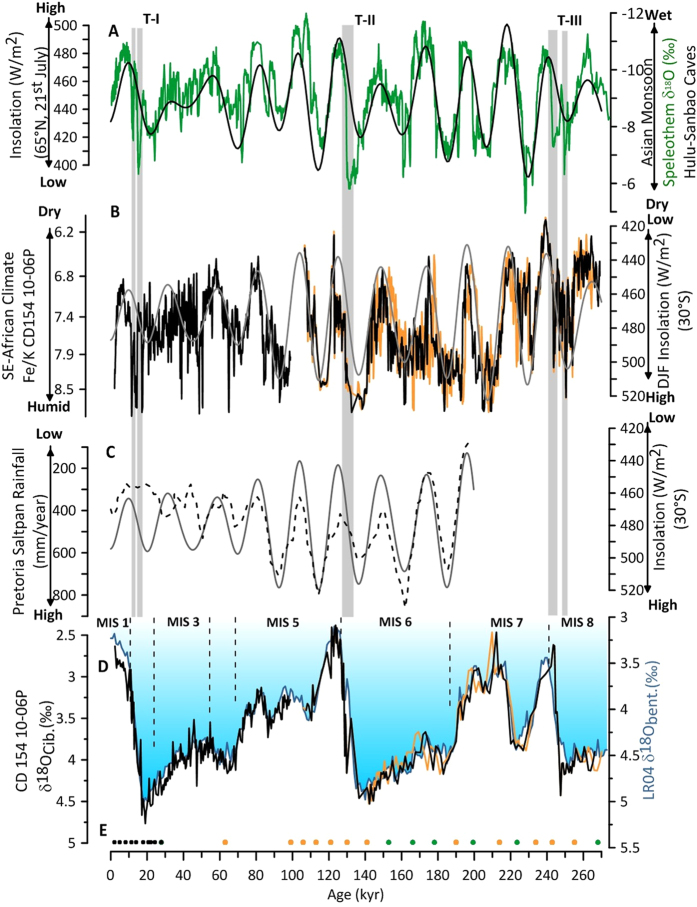
Long-term climate variability in eastern South Africa over the past 270,000 years. (**A**) δ^18^O splice from Chinese speleothems^40^,^41^,^44^, (green) as presented in Barker *et al.*, 2011(ref. 64) showing synchronous variability of the East Asian Monsoon with Northern Hemisphere summer insolation at 65° N. Underlying grey bars indicate glacial-interglacial Terminations (T) (**B**) Fe/K of CD 154 10-06P (black, 5 point running mean, fine-tuned age model) indicating more humid KwaZulu-Natal climate in accordance with varying austral (DJF) summer insolation at 30 °S. Light orange record shows initial age model based on LR04 tuning (**C**) Pretoria Saltpan Rainfall record (mm/year)^16^ with December insolation at 30 °S (**D**) Benthic foraminiferal (*Cibicidoides* spp.) δ^18^O record from CD154-10-06P (black, fine-tuned age model; light orange initial age model), reflecting global ice volume variability and local deep-water conditions, in comparison with global benthic stack LR04 (blue). Marine isotope stages (MIS) indicated (**E**) Age control points for CD154-10-06P, including radiocarbon dates, (black), tuning of the foraminiferal δ^18^O record (light orange) and δ^18^O splice from Chinese speleothems on precession phase (green).

An initial chronology for CD154-10-06P is based on ten accelerator mass spectrometry radiocarbon dates in the upper part of the record (Methods). Beyond the limits of the radiocarbon method we established an initial age model based on the graphic correlation of the benthic δ^18^O (*Cibicidoides* spp.) record of core CD154-10-06P to the global benthic stack LR04^30^, (Fig. 2D). The benthic δ^18^O record reflects the combined influence of ambient deep-water temperature variability in the southwest Indian Ocean, global ice volume and local salinity changes and shows a good fit with long-term variability in the benthic δ^18^O stack of 57 globally distributed sites. The sediment core spans a period of approximately the last 270,000 years and displays an average sedimentation rate of 4 cm kyr^−1^ (SF.4). On the basis of this initial age modelling approach, our Fe/K record shows clear orbital-scale oscillations (Fig. 2B) between high ratios (indicating more humid conditions) and lower values (pointing towards drier conditions in eastern South Africa). Spectral analysis of the Fe/K record of CD154-10-06P reveals the highest spectral power in the 23-kyr band (99% confidence level (CL)), which is persistent throughout the past 270,000 years (SF.4, 5; SI). Orbital-timescale variations in the Fe/K record of CD154-10-06P can be linked to changes in austral summer insolation (December-January-February; (DJF)) over South Africa (30°S), and reflect the 23-kyr periodicity of orbital precession, which dominates local summer insolation changes at low latitudes^31^ (Fig. 2B). Assuming that sufficient moisture is available, stronger summer insolation may intensify atmospheric convection over eastern South African and result in higher precipitation.

**Figure 3:**
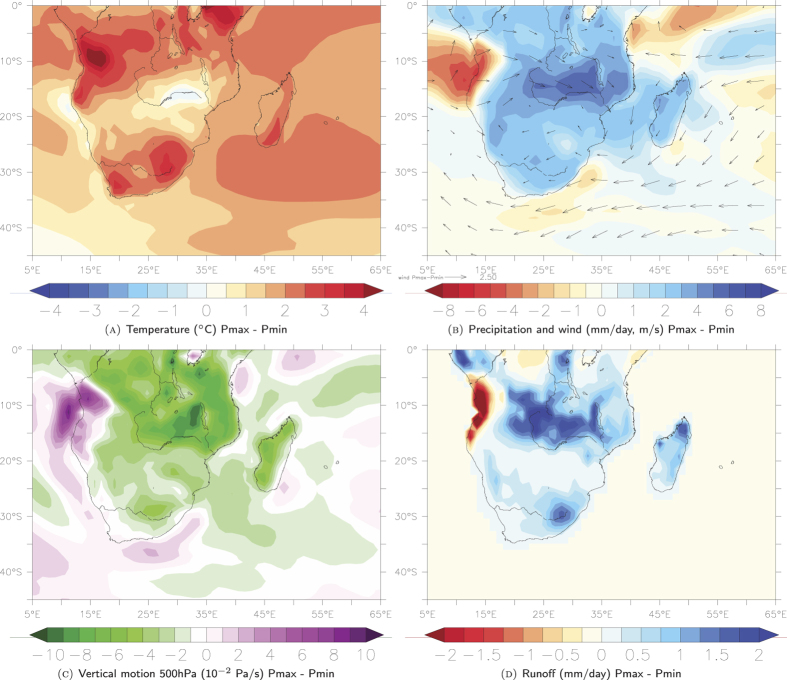
Results of idealised sensitivity precession experiments using high-resolution fully coupled ocean-atmosphere model EC-Earth. Figures were created using the model output and the program Ferret, version 6.82 (version for Mac). Ferret is a tool developed by NOAA, http://www.ferret.noaa.gov/Ferret/. (**A**) Temperature in Celcius for December, January, February (DJF). Difference between maximum and minimum precession. (**B**) Precipitation in mm/day and wind in m/s for DJF. Difference between maximum and minimum precession. (**C**) Vertical motion for difference between maximum and minimum precession at 500 hPa (roughly 5km height) is given in 10^−2^ Pa/s. Negative values indicate upward motion, positive values indicate downward motion (**D**) Surface runoff over land in mm/day for DJF. Difference between maximum and minimum precession. (Results for the individual Pmax and Pmin runs can be found in SF.7).

In order to test this assumption we re-analyse precession only sensitivity experiments (altering the seasonal and latitudinal distribution of solar insolation) from Bosmans *et al.*, 2015 (ref. 32) using a state-of-the-art high resolution fully coupled ocean-atmosphere model EC-Earth (Methods). We use two idealised scenarios: one where summer solstice occurs near perihelion (minimum precession, Pmin) and one where summer solstice occurs near aphelion (maximum precession, Pmax). Our model results show that during Pmax, higher DJF insolation causes higher temperatures over the Southern Hemisphere, especially over land (Fig. 3A). Related to the changes in temperature, surface pressure is lower over land (SF.6). Specifically, lower pressure during Pmax compared to Pmin over South Africa and higher surface pressure over the southern Indian Ocean results in stronger easterly surface winds blowing towards eastern South Africa (Fig. 3B). Over land, convection (upward motion) is increased (Fig. 3C). Precipitation over nearly all of southern Africa is higher during Pmax (Fig. 3B), as is net precipitation (precipitation minus evaporation, SF.6) supporting our proxy-based results (Fig. 2). The signal is particularly pronounced in the KZN and Eastern Cape province region, compared to others further north or west, which results in higher surface runoff in the KZN and Eastern Cape province during Pmax influencing core sites adjacent to land in the SW Indian Ocean (Fig. 3D).

Increased (net) precipitation and runoff are only partly due to increased local evaporation. We computed, to a first order approximation, the precipitation as well as evaporation increase over the area 25S-35S; 25E-35E (land points only ;(dP-dE)/dP) where ‘d’ indicates the Pmax-Pmin difference). The results indicate that during DJF 67% of the precipitation increase originates from outside this land area. Precipitation increase is therefore more likely a result of increased humidity (not shown) and wind. Moisture transport (the product of specific humidity and horizontal winds vertically integrated over the whole atmospheric column) shows that during Pmax, moisture transport is in a more northeasterly direction over the coast of Mozambique. Overall, there is more northerly to northeasterly moisture transport over eastern South Africa bringing a moist and more tropical air mass over the region (SF.6), thereby leading to increased precipitation and runoff in this area. This is mainly due to the dynamic part of the moisture transport (due to wind change, not shown), which indicates increased moisture transport into eastern South Africa during Pmax. This explains, in combination with the observed regional intensification of convection, the increased precipitation and subsequently, higher runoff in KZN (Fig. 3B–D).

Our record of long-term climate variability over eastern South Africa is consistent with other regional records that track Southern Hemisphere summer insolation changes^14^,^16^,^33^. In particular, the correspondence between the CD154-10-06P record and the Pretoria Saltpan (Lake Tswaing) time series^16^, potentially a more direct indication of terrestrial summer precipitation, supports the assumption that the wider South African climate responded to changes in orbitally modulated insolation (Fig. 2). Sensitivity experiments using CCSM3 provide further confirmation of a regionally consistent hydrological response to summer insolation changes in eastern South Africa^8^. While the in Otto Bliesner *et al.*, 2014 (ref. 8) presented results are consistent with our precession-only forced model ones in the region of the Zambezi catchment, Madagascar and eastern South Africa (Fig. 3) we find deviations in the precipitation pattern in southeast equatorial Africa. This discrepancy might be related to the different applied boundary conditions in these simulations^8^. A variety of records covering the late Pleistocene across the Southern Hemisphere (Fig. 1) indicate increased precipitation during Southern Hemisphere summer insolation maxima when the perihelion occurs close to the summer solstice^15^,^34^,^35^,^36^,^37^,^38^,^39^ and thus exhibit an interhemispheric anti-phased relationship with Northern Hemisphere records in the precession band^11^,^12^,^40^,^41^.

The present day climate in subtropical South Africa shows strong seasonality with a distinct winter rainfall zone in the far west and southwest, and mainly summer rainfall elsewhere. Based on a hyrax-midden record from the Namib Desert it has been suggested that the southwest African hydroclimate (winter rainfall zone) was wetter during the Southern Hemisphere summer insolation minimum during the early Holocene (thus suggesting the opposite precipitation pattern to the CD154-10-06P record) and responded in phase with the declining Northern Hemisphere summer insolation across the Holocene^42^. Conversely, a recent record showed an in-phase relation of the winter rainfall zone with Southern Hemisphere insolation maxima which would imply reduced seasonality within the South African rainfall zones on precession timescales^14^ (Fig.1). Previous studies also recognised shifts in the South African hydroclimate on glacial-interglacial timescales but are mostly limited to the winter rainfall zone^13^,^14^,^17^. The record presented here is clearly dominated by a 23–19 kyr cycle signal and does not display any significant 100*-*kyr glacial-interglacial variability (Fig. 2, SF.4). The latter contrasts recent findings arguing that the significance of direct insolation forcing on the summer rainfall zone only developed during the Holocene^43^. This might imply that the southern African summer rainfall zone was rather heterogeneous with distinct areas responding predominantly to precession forcing (Fig. 3) whereas in others the precipitation amount was potentially more tightly linked to changes in the Indian Ocean temperatures during MIS 3 and 2 (ref. 43).

### Millennial-scale climate variability in the KwaZulu-Natal province

The speleothem δ^18^O records from eastern China are thought to represent changes in the proportion of low δ^18^O (summer) rainfall within annual totals displaying East Asian Monsoon (EAM) variability^44^ (SI). The significant negative correlation (PearsonT R^2^ = 0.25; bootstrap error uncertainty 0.04; 0.5 at CL 95%) between precipitation in East Asia versus that in eastern South Africa, as implied by the records shown in Fig. 2, suggests an anti-phase relationship between Northern and Southern Hemisphere rainfall systems at the precession timescale. We therefore take advantage of the precise absolute dating of the speleothem record to refine the age-scale of CD154–10-06P by synchronising the records at the precession band (Fig. 2). This fine-tuning approach is supported by the continued high level of synchronicity between the benthic δ^18^O record of core CD154 10-06P and the LR04 stack (PearsonT R^2^ = 0.92; bootstrap error uncertainty 0.86; 0.95 at CL 95%), (Fig. 2). The average absolute age difference between the initial and the resulting fine-tuned age model is only ~400 years (1σ = 1.47 kyr; SI).

**Figure 4:**
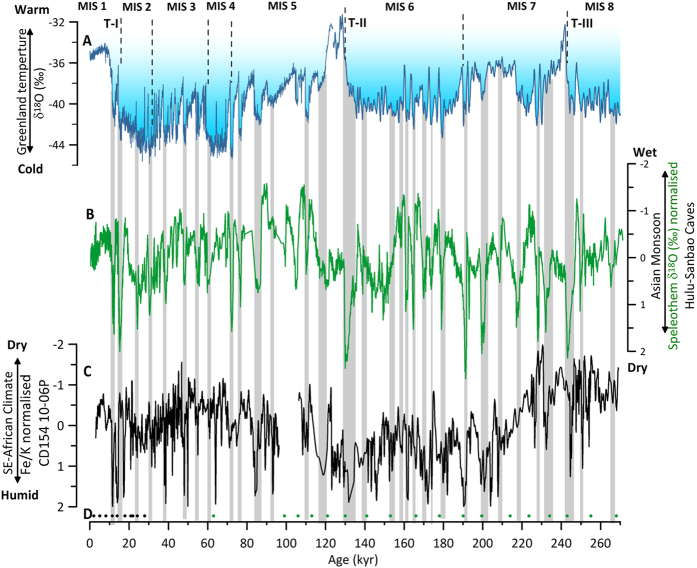
Millennial-scale climate variability in eastern South Africa over the past 270,000 years. (**A**) δ^18^O record from Greenland ice core NGRIP^70^, (black, Speleo-age model presented in Barker *et al.*, 2011 (ref. 64) displaying abrupt temperature variability in the North Atlantic) (**B**) Normalised δ^18^O splice from Chinese speleothems^40^,^41^,^44^ (green) as presented in Barker *et al.*, 2011 (ref. 64) showing synchronous variability of the EAM with Northern Hemisphere climate variability. Grey bars indicate synchronicity between Greenland Stadials, weak EAM intervals and humid phases in eastern South Africa (**C**) Normalised Fe/K record of CD154 10-06P (7 point running mean) (**D**) Age control points for CD154-10-06P, including radiocarbon dates, (black), tuning of the foraminiferal δ^18^O record and δ^18^O splice from Chinese speleothems on precession phase (green).

It has been demonstrated that abrupt changes to more humid conditions in southeast Africa and the Eastern Cape province were associated with Greenland stadials and periods of weak EAM, resulting in an anti-phasing between rainfall variability in the two hemispheres on a millennial timescale over the last glacial cycle^19^,^33^. After fine-tuning the Fe/K record of core CD154 10-06P to the Chinese speleothem record in the precession-band we find a similar relationship between millennial-scale features in our record and the speleothem record over the past 270,00 years (Fig. 4). Almost every abrupt weak EAM event can be matched with a humid interval in the eastern South African record, which implies a pervasive interhemispheric teleconnection (Fig. 4). Especially pronounced are the humid conditions in the KZN during, for example, glacial terminations T-III and T-II, which potentially document an amplified hydrological signal in the region at these times due to the combined effects of orbital precession maxima and abrupt climate change during terminal Heinrich stadials (Fig. 4). However, we note a divergence from this mode of interhemispheric teleconnection during the last deglaciation Heinrich Stadial 1, when low Fe/K values suggest rather dry conditions in eastern South Africa (Fig. 4).

The interhemispheric linkage in precipitation patterns on millennial timescales is thought to be caused by latitudinal shifts in the position of the ITCZ and subtropical anticyclones in response to variations in the mode of Atlantic overturning and North Atlantic temperature^45^. Such changes could be propagated to the tropical latitudes via rearrangements of atmospheric circulation^46^. The tropical mean circulation responds to the Northern Hemisphere cooling (and Southern Hemisphere warming) by generating anomalous energy transport from the Southern Hemisphere to the Northern Hemisphere. This energy transport is accomplished by a reorganisation of the mean Hadley circulation, involving an anomalous northward cross-equatorial flow in the upper branch accompanied by an anomalous southward flow in the lower branch^47^. This produces a southward shift of the equatorial and near-equatorial precipitation belts associated with the ITCZ^48^. While the ITCZ is likely to have shifted southward during North Atlantic cold events, directly impacting records north of our core location, e.g.^33^, it probably did not reach the KZN region directly. However, even if the ITCZ did not shift very far south during North Atlantic cold events, there would have been a southward shift of the subtropical anticyclones bringing KZN more often under the influence of onshore moist easterly flow from the SW Indian Ocean and hence becoming wetter^49^.

In addition to the regional subtropical anticyclones, summer rainfall along the coast of KZN and the Eastern Cape province is strongly influenced by the warm Agulhas Current^50^ which provides low-level moisture to facilitate the occurrence of deep convection and rainfall as the onshore flow reaches the coast^51^. Conversely, atmospheric model simulations^52^ indicate that a cooler Agulhas Current leads to a cooler and drier air mass over southeast Africa and a rainfall decrease. We suggest that the precipitation increase may partly arise from a warming in the Agulhas Current as a feature of the bipolar seesaw during North Atlantic cooling. Mg/Ca-derived upper ocean temperatures reconstructions of core CD154 17-17 K (Fig. 1) partly supports this assumption, indicating warming in the current during most Northern Hemisphere cooling episodes, being consistent with the bipolar seesaw theory^53^. Increased upper ocean temperatures may have produced sufficient precipitation over eastern South Africa. Likewise, due to the established build-up of heat in the Southern Hemisphere during times of reduced AMOC, the atmosphere is warmer and moister, and hence favourable for more convective precipitation.

In contrast to our findings and previous work from offshore Eastern Cape province^19^, based on a recent compilation of pollen records^43^ it has argued that eastern and southern Africa was apparently under dry conditions during cold anomalies in the Northern Hemisphere. The observed pattern was linked to cooling in the western Indian Ocean SSTs, which was caused by increased upwelling off the coast of Somalia, and the Arabian Peninsula via an expansion of the Mediterranean-Arabian anticyclone as shown by Otto-Bliesner *et al.*, 2014 (ref. 8). However, it should be noted that while cooler western Indian Ocean SSTs both north and south of the equator might explain a reduction in precipitation in southeastern equatorial Africa during Northern Hemisphere cold anomalies, the same sensitivity experiment demonstrates that southwest Indian Ocean temperatures (along the Agulhas Current) are increased instead^8^. Simultaneously precipitation is elevated in southern Mozambique and South Africa^8^ thus supporting our findings.

A growing number of records from either side of the Equator document reduced rainfall in the Northern Hemisphere^40^,^54^,^55^,^56^ and a concomitant strengthening rainfall in the Southern Hemisphere counterpart^34^,^36^,^57^,^58^ during times of Northern Hemisphere cooling episodes (Fig. 1). However, none of these records reflect the palaeohydrology in the eastern South African region, which highlights the importance of our new record.

Through the combined effect of regional insolation changes caused by orbital precession and the effects associated with high-latitude abrupt climate forcing, eastern South Africa potentially offered favourable environmental conditions compared to the rest of the African continent, thereby permitting early human settlement in that region. How climate variability potentially influenced settlement and evolution of *Homo sapiens* in that area during the Middle Stone Age remains to be shown due to the current scarcity of well-dated archaeological sites. The findings presented here confirm recent CCSM3-based results^8^ for past climate variability in eastern South Africa - an area from which detailed long-term palaeo-archives suitable for comparison with model simulations and archaeological evidence were previously unavailable. The link between climate, population growth/settlement and innovation is also important for us today. While recurrent shifts between long-term droughts and humid phases in eastern South Africa during the past 270,000 years arose from natural causes, climate models project that this region will undergo progressive aridification in the future as part of a general drying and poleward expansion of the subtropical dry zones driven by the human-induced rise in GHGs^18^.

## Methods

### Age model

The age model for the upper core sections was developed using ten ^14^C accelerator mass spectrometer (AMS) dates measured from samples containing approximately 1000 tests of *G. ruber* (>250–315 μm) and has been previously presented in^59^. Radiocarbon measurements were made at the Natural Environment Research Council (NERC) Radiocarbon Laboratory (Supplementary Table 1). The radiocarbon ages were converted into calendar years using the Marine09 data set^60^ with the global mean reservoir correction of (R) 405 years^61^. The core chronology was constructed using the statistical package BChron^62^,^63^ using a Bayesian approach to calculate the 95% (2σ) uncertainty on the calibrated ages (Supplementary Table 1) and the 95% probability envelope for the time period studied (SF.2) In the range of the ^14^C dates (1.9–27.9 ka) average sedimentation rates of ~ 4.0 cm ka^−1^ (4.8–1.9 cm ka^−1^) and a sample integration of ~300 years for every 1 cm sample is implied. Beyond the limits of the radiocarbon method graphic tuning of the benthic δ^18^O record to the global benthic stack LR04^30^, was used to establish the initial age model (Fig. 2). To further fine-tune the age model we visually match common transitions within the Fe/K ratio and the speleothem record from Chinese Caves, Hulu as presented in^64^ on the precession band (Fig. 2). Ages between each age control point were estimated by linear interpolation. However, it was not possible to establish a continuous age model on the turbidite adapted depth scale (SF.1; Fig. 2) of the core as the event caused sediment erosion in that interval evident through the absence of half a precession cycle in the Fe/K record during MIS 5 c/d (Fig. 2). In order to adapt for the time gap (~7 kyr), two additional tuning points were used (Fig. 2).

### Benthic Oxygen isotopes

In this study, three to ten tests of epibenthic foraminiferal species *Cibicidoides mundulus* [Brady, Parker & Jones, 1888] and/or *Cibicidoides wuellerstorfi* [Schwager, 1866], (summarised as *Cibicidoides* spp.) were picked in core CD154 10-06P from the 250–315 μm size fraction or <250 μm in sections of low abundance of *Cibicidoides* spp. A study by Ruiz *et al.*, 2013 (ref. 65) showed that shell size does not influence the isotopic composition of *Cibicidoides* spp. If enough material was available, measurements were performed at 2 cm intervals until 550 cm core depth and thereafter every 4 cm. Stable isotopes were measured using either a ThermoFinnigan MAT 252 mass spectrometer linked online to a Carbo Kiel-II carbonate preparation device (long-term external precision is 0.06% for δ^18^O and 0.02% δ^13^C) or a Thermo Scientific Delta V Advantage mass spectrometer coupled with a Gas Bench III automated preparation device (long-term external precision is 0.08% for δ^18^O and 0.06% δ^13^C) depending on the sample size. The stable isotope measurements were expressed relative to the Vienna Peedee Belemnite scale (VPDB) through calibration with the NBS‐19 carbonate standard.

### Elemental records

Core scanning was performed using the ITRAX™ XRF Core scanner at the British Ocean Core Research Facility (BOSCORF, Southampton). Measurements were made at 0.1 cm resolution with a count time of 30 s, at 30 kV and 50 mA for the XRF scan. Additionally, for validation of the XRF scans, major and trace elements were analysed on bulk sediment for a discreet subset of samples (n = 18). Analysis of the bulk sediment samples was performed using a Thermo X Series II inductively coupled plasma mass spectrometer (ICP-MS). Approximately 0.1 g of freeze-dried and homogenized sediment was ignited in a furnace at 900 °C (58) loss on ignition values. Whole-sediment major element concentrations were obtained following Li metaborate fusion. The internationally recognized standard JB-1A was run alongside the sample batch. Long-term relative standard deviations show precision of 1–2% for major trace elements for JB-1A. XRF scanning-derived Fe/K ratios of core CD154 10-06P were calibrated to absolute bulk elemental concentrations following the approach of Ziegler *et al.*, 2013 (ref. 19) using the equation: Fe/K_bulk_ = (0.1456*Fe/K_XRF_) + 2.0533 (Fig. 2). XRF scanning-derived counts/ratios are displayed on logarithmic scale within this work^66^.

### Model description

Model results are based on precession experiments with the state-of-the-art high resolution fully coupled ocean-atmosphere model EC-Earth^67^. Version 2.2 uses the Integrated Forecast System (IFS) of the European Centre for Medium-Range Weather Forecast (ECMWF), cycle 31R1, for the atmosphere with a resolution of T159 (~1.125degree). The ocean consists of the Nucleus for European Modelling of the Ocean^68^,^69^ (NEMO, run at ~1degree resolution). Here we use two idealized precession experiments, one where summer solstice occurs near perihelion (minimum precession, Pmin) and one where summer solstice occurs near aphelion (maximum precession, Pmax). All other boundary conditions (e.g. land ice, greenhouse gasses, vegetation) are fixed at pre-industrial levels. The precession experiments and resulting insolation changes are described in more detail in Bosmans *et al.*, 2015(ref. 32).

## Additional Information

**How to cite this article**: Simon, M. H. *et al.* Eastern South African hydroclimate over the past 270,000 years. *Sci. Rep.* **5**, 18153; doi: 10.1038/srep18153 (2015).

## Supplementary Material

Supplementary Information
