# Supplementary material for: Eastern South African hydroclimate over the past 270,000 years
Source: Sci Rep. 2015 Dec 21;5:18153. doi: 10.1038/srep18153 (PMC4685309; doi:10.1038/srep18153)
Supplement: Supplementary Information [file srep18153-s1.pdf]

Supplementary Information

Eastern South African hydroclimate over the past 270,000 years

Margit H. Simon[a,*], Martin Ziegler[a,b], Joyce Bosmans[b], Stephen Barker[a], Chris J.C. Reason[c] and Ian R. Hall[a]

[a]*School of Earth and Ocean Sciences, Cardiff University, Cardiff, CF10 3AT, United Kingdom*

[b]*Faculty of Geosciences, Utrecht University, 3584 CD Utrecht, Netherlands*

[c]*Department of Oceanography, University of Cape Town, South Africa*

[*] *present address: Uni Research Climate, Allegaten 55 and Bjerknes Centre for Climate Research, Allegaten 70, 5007 Bergen, Norway.*

Age model

Marine sediment core CD154 10-06P recovered 969 cm of marine mud mainly composed of foraminiferal ooze. The core was sampled at 1 cm intervals, the wet sediment was weighed, disaggregated on a rotating wheel for approximately 24 hours, washed over a 63 µm sieve using fine water spray and dried in the oven at 40 °C.

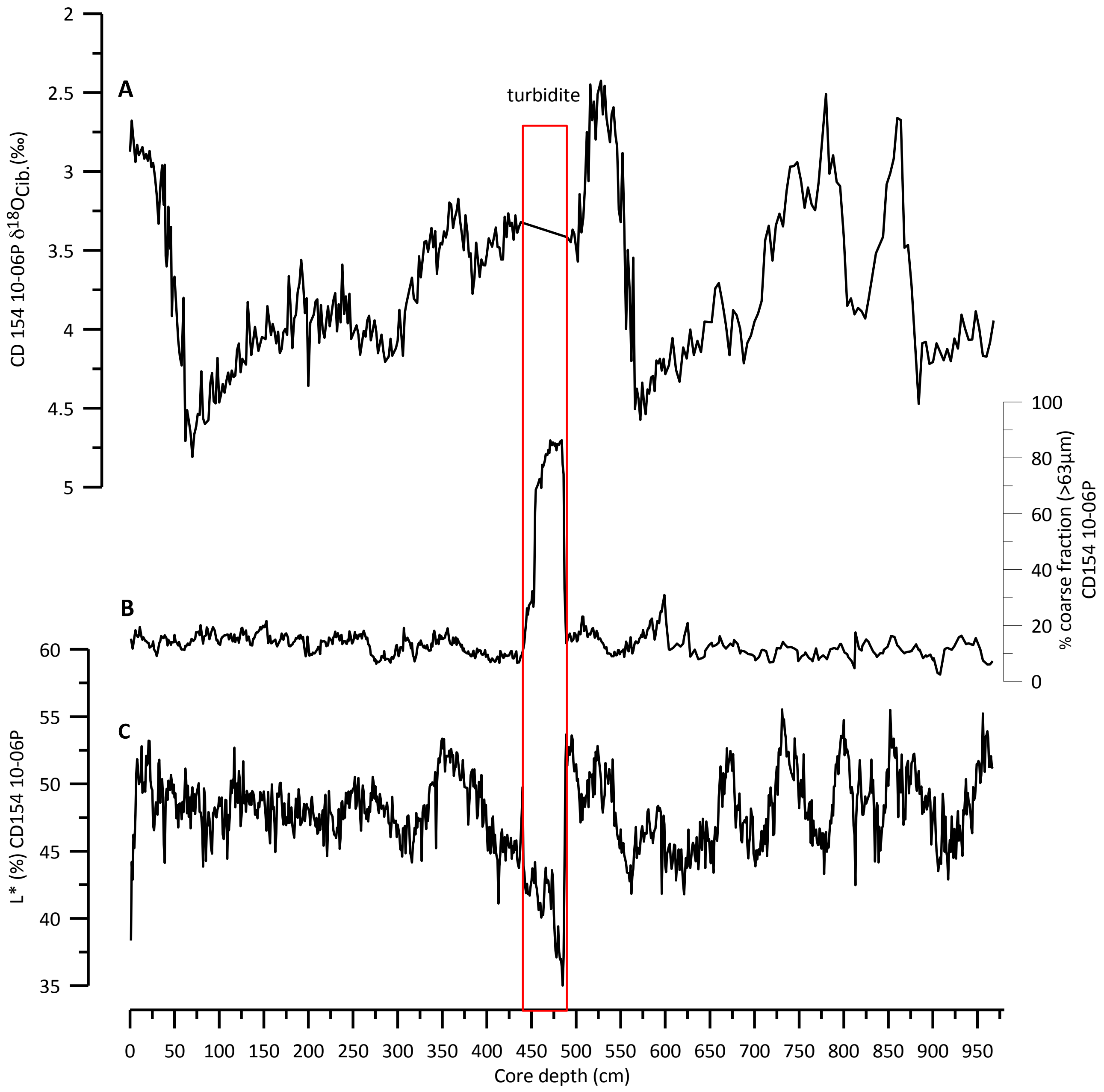

Supplementary Figure 1: Core depth (cm) of marine sediment core CD154 10-06P with the interval highlighted (red box) which is affected by turbidite (A) Benthic isotope record of CD154 10-06P (B) % coarse fraction (C) L* (light reflectance) record.

A turbidite was detected during sampling in the interval 440-490 cm core depth through visual inspection as samples in that interval were noticeably sandy with grading features containing no benthic foraminifera (Supplementary Figure 1). Moreover, a rapid increase in the coarse fraction weight % in the affected interval confirms the assumption (Supplementary Figure 1). A sharp excursion in the L* reflectance of the record further confirmed the feature (Supplementary Figure 1). As such, the identified turbidite interval (~50 cm) was not used for the paleoceanographic records. The age model construction was performed on the new adjusted composite depth of the core.

Supplementary Table 1 ^{14}C dates for sediment core CD154-10-06P

Depth (cm) CD154 10-06P	Species	^{14}C age BP (yr)	Error +/- 1σ (radiocarbon yrs BP)	2σ confidence age interval Lower limit (ka BP)	2σ confidence age interval Mid-point (ka BP)	2σ confidence age interval Upper limit (ka BP)	Laboratory Code
0-1 cm	*G.ruber*	2359	35	1.88	1.98	2.09	SUERC-45072
14-15 cm	*G.ruber*	4774	35	4.89	5.03	5.20	SUERC-45075
26-27 cm	*G.ruber*	7681	40	8.02	8.14	8.26	SUERC-45076
40-41 cm	*G.ruber*	10409	49	11.26	11.48	11.71	SUERC-45077
51-52 cm	*G.ruber*	12403	63	13.70	13.85	14.01	SUERC-45078
64-65 cm	*G.ruber*	15082	89	17.50	17.85	18.40	SUERC-45079
78-79 cm	*G.ruber*	17863	132	20.37	20.79	21.29	SUERC-45080
83-84 cm	*G.ruber*	18786	148	21.51	21.91	22.31	SUERC-45081
92-93cm	*G.ruber*	20682	191	23.69	24.19	24.76	SUERC-45082
99-100 cm	*G.ruber*	23498	273	26.97	27.90	28.48	SUERC-45085

Supplementary Table 2: Age control points for the age model of sediment record CD154-10-06P

**Radiocarbon dates**		**Tuning of benthic δ^{18}O of CD154-10-06P to LR04**[1]		**Additional tuning of Fe/K of CD154-10-06P to Chinese speleothem δ^{18}O splice**	
Depth (cm)	Age (kyr)	Depth (cm)	Age (kyr)	Depth (cm)	Age (kyr)
0.5	1.98	281	63	459	113
14.5	5.03	439	99	567	153
27.5	8.14	440	106	602	166
40.5	11.48	459	113	631	178
51.5	13.85	463	121	702	199.5
64.5	17.85	513	130	755	223.5
78.5	20.79	518	141	911	268
83.5	21.91	658	190		
92.5	24.195	736	214		
99.5	27.903	795	234		

		807	243		
		879	255		

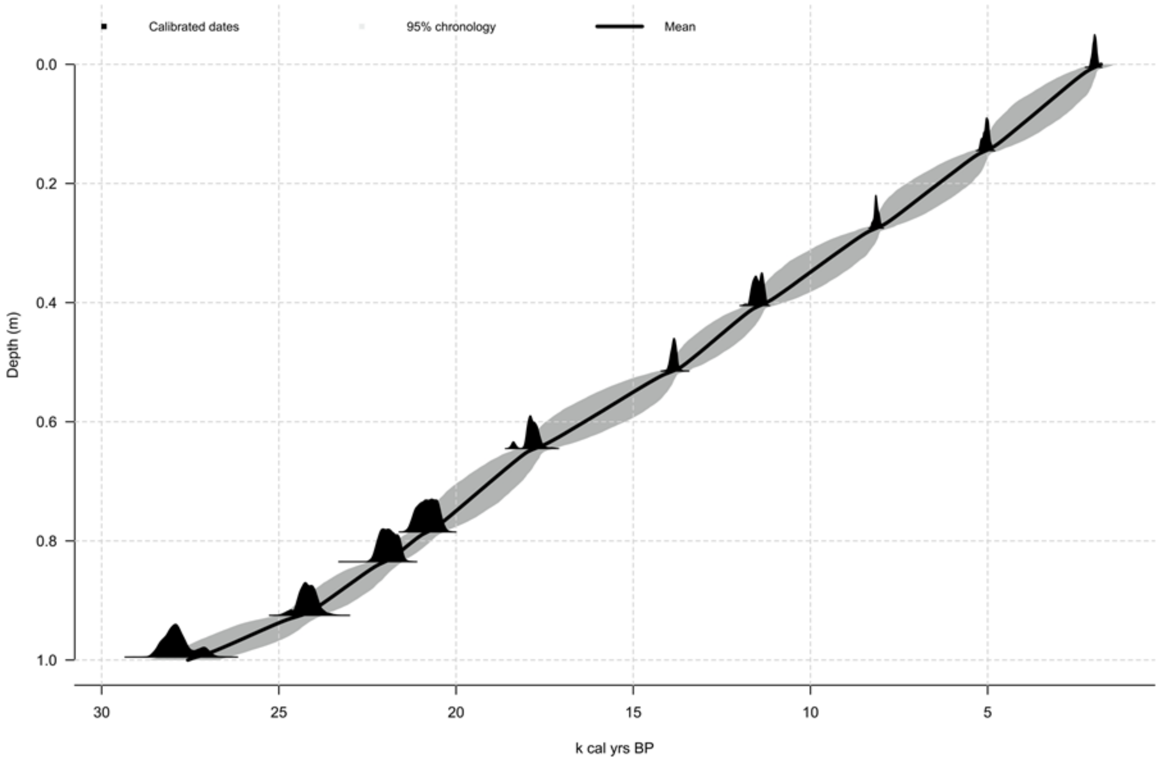

Supplementary Figure 2:The Bayesian age-model obtained by Bchron (black) for the top 100cm of CD154 10-06P incorporating a local reservoir uncertainty of 405 years (ΔR=0). Each date is represented by the probability distribution of the intersection between the radiocarbon ages at those depths and the Marine09 calibration curve. The grey shaded area indicates the 95% probability based on the calibrated dates using the Bayesian statistical package Bchron[2].

Pearson Correlation analysis

In this study, time-series correlation was performed using the PearsonT programme[3]. In PearsonT, the Pearson's correlation coefficient is estimated employing a nonparametric stationary bootstrap confidence interval with an average block length proportional to the maximum estimated persistence time of the data[3].

Spectral analysis

Power spectra were calculated with the REDFIT software[4] employing a Welch-Overlapped-Segment-Averaging procedure, a Hanning window and four overlapping (50%) segments; red-noise boundaries were estimated as upper 80, 90 and 95% chi-square limits of a fitted first order autoregressive process AR(1). According to Mann and Lees [5] a large proportion of the spectra of climatic-proxy data have backgrounds which correspond to a first order

autoregressive process AR(1). Cross-spectral analysis to compute phase estimates and coherences were calculated with the SPECTRUM software[6]. Gaussian filtering was performed using the software Analyseries[7].

The Fe/K record of CD154-10-06P reveals highest spectral power in the 23-kyr band of precession (confidence level (CL) 99%), (Supplementary Figure 4) which is present throughout the past 270 kyr, however not consistently with the same power through time (Supplementary Figure 5). Periodicities centred around 19-23-kyr (precession) were found to stand out over the background noise with high power at above 95% CL but are limited to 60-270 kyr, albeit extending into the 'cone of influence' where possible edge effects may be increasingly important (Supplementary Figure 5). Eccentricity modulates precession by changing the shape of the Earth's orbit and that controls how effective precession is in modulating the seasons by controlling how close to the sun the perihelion is[8,9]. As eccentricity moves towards a value of 0 (circular) the effect of precession is reduced. The connection with regional insolation holds throughout the record, but is less clear during the last glacial period (Supplementary Figure 4). We propose that this diminished forcing by insolation in our record is due to the lower amplitude of precessional cycles in those intervals due to low eccentricity since 100 kyr[10].

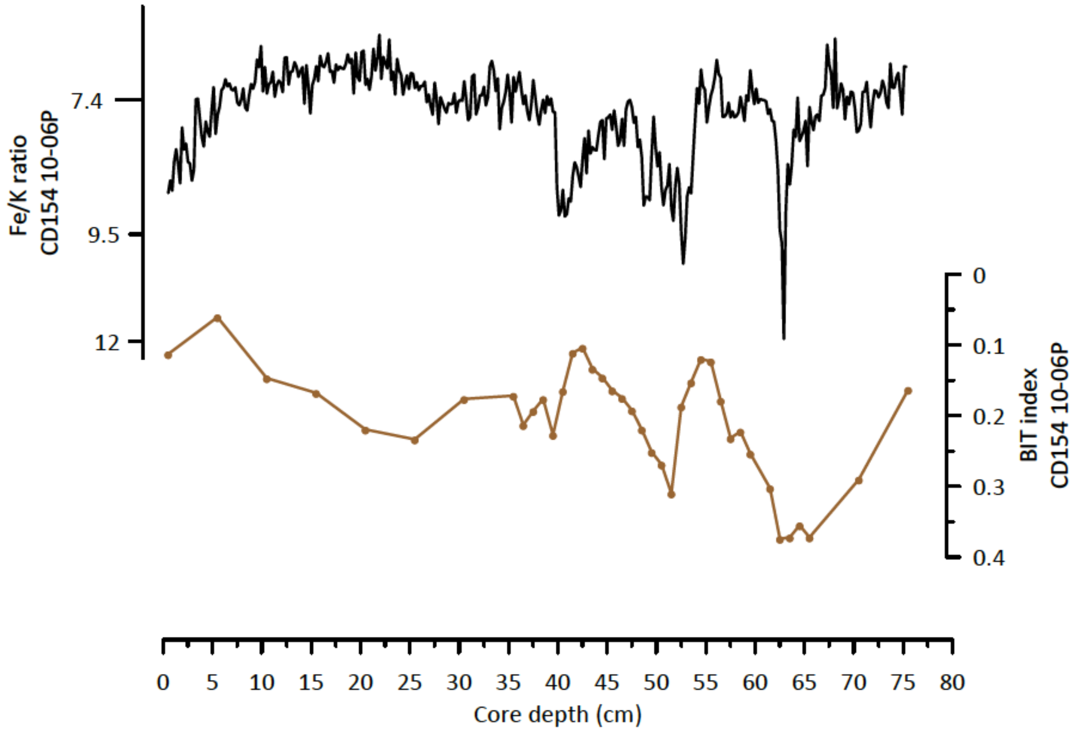

Supplementary Figure 3: (A) Fe/K ratio and (B) BIT index CD154 10-06P

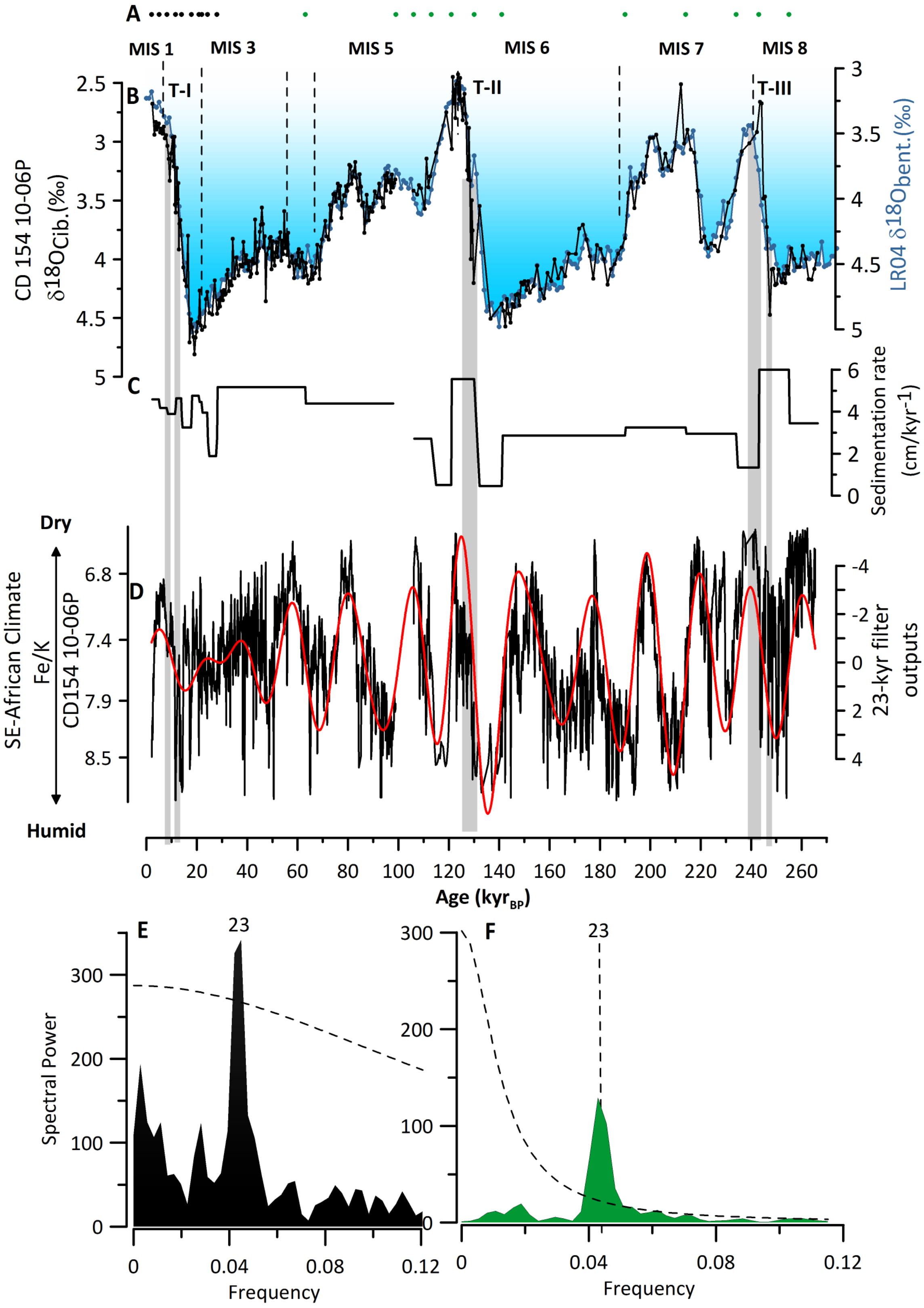

Supplementary Figure 4: Initial Age model for core CD 154 10-06P. (A) Age control points for CD154-10-06P, including radiocarbon dates, (black), tuning of the foraminiferal $\delta^{18}O$ record to LR04 (green) (B) Benthic foraminiferal (*Cibicidoides* spp.) $\delta^{18}O$ record from CD154-10-06P (black), reflecting global ice volume variability and local deep-water conditions, in comparison with global benthic stack LR04 (blue). Marine isotope stages

(MIS) indicated, Underlying grey bars indicate glacial-interglacial Terminations (T), (C) Sedimentation rates in cm kyr-[1] (D) Fe/K of CD 154 10-06P (black, 5 point running mean) with 23-kyr Gaussian filter on top (red) (E) Power spectra calculated with the REDFIT-sofware for Fe/K record of core CD154 10-06P (black) and (F) Chinese speleothems $\delta^{18}O$ record (green), red noise boundaries were estimated as upper 99% chi-squared limits of a fitted AR1 process. Bandwidth is 0.0186. Precession band (23-kyr) is highlighted

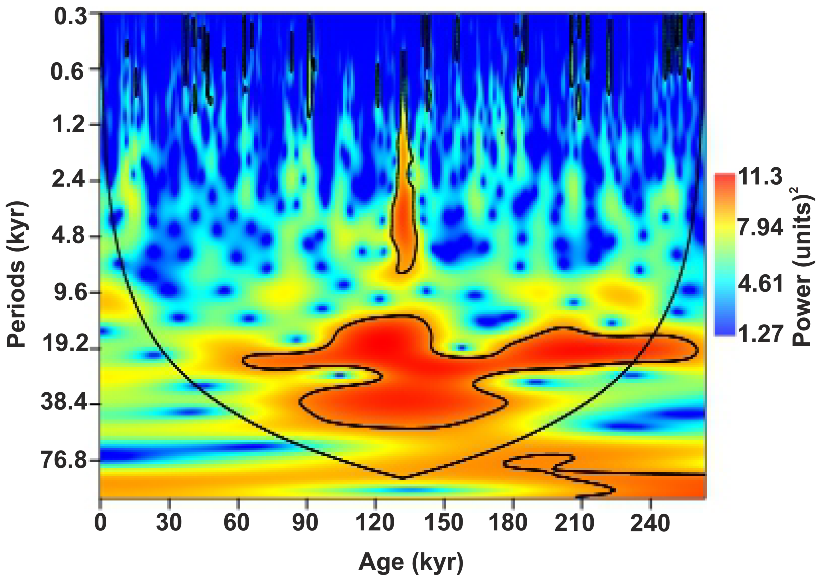

Supplementary Figure 5: Wavelet analysis on the 150 year linear interpolated Fe/K record from CD154 10-06P using PAST software[11]. Colours denote power above red-noise. The black outline indicates confidence level of 95% assuming a red-noise model. Vertical axis in the plot is a logarithmic size scale (base 2). Signal power (squared correlation strength with the scaled mother wavelet) is shown in colour. “Cone of influence” shows the region where boundary effects are present.

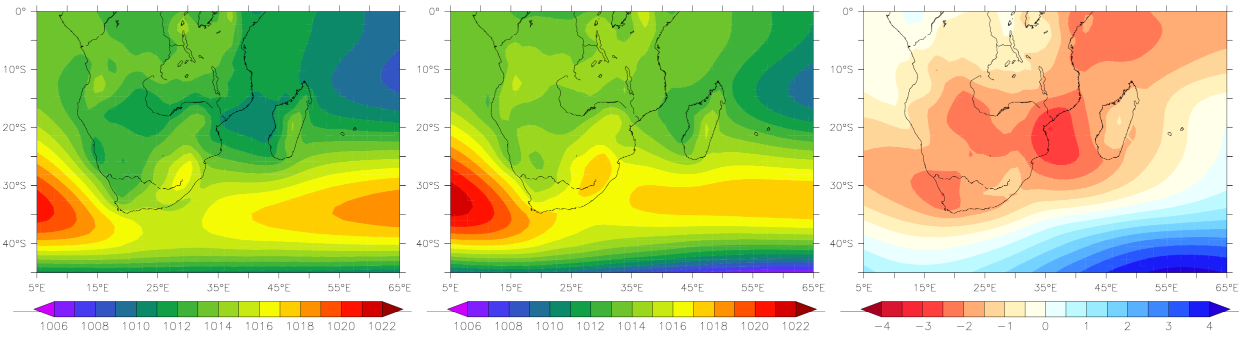

(A) Sea level pressure (hPa) DJF Pmax (B) Sea level pressure (hPa) DJF Pmin (C) Sea level pressure (hPa) Pmax - Pmin

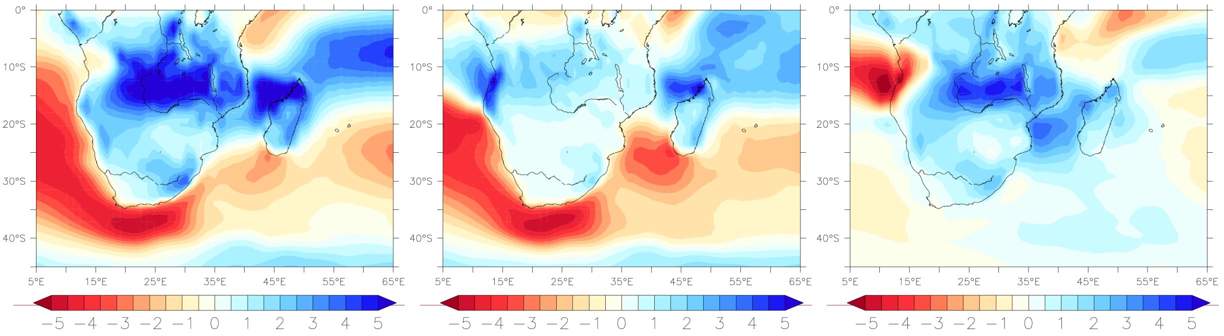

(D) Net precipitation (mm/day) DJF Pmax (E) Net precipitation (mm/day) DJF Pmin (F) Net precipitation (mm/day) Pmax - Pmin

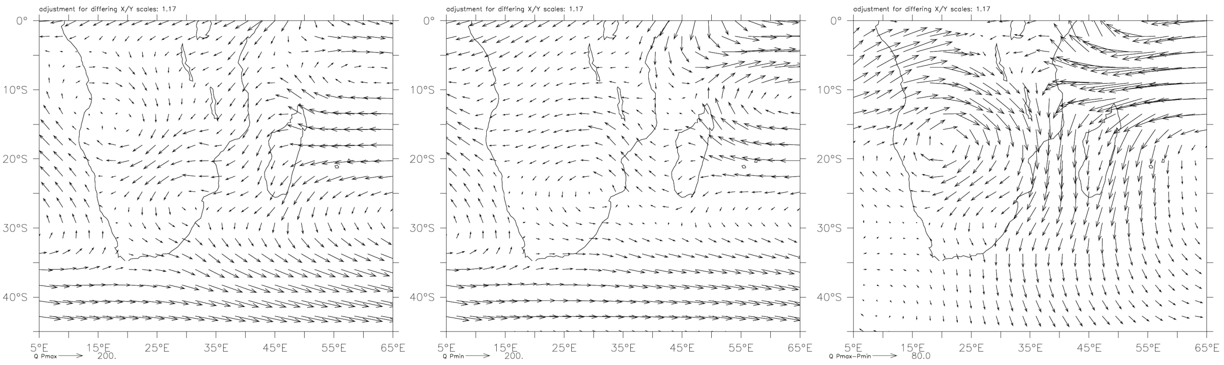

(G) Moisture transport (kg/(ms)) DJF Pmax (H) Moisture transport (kg/(ms)) DJF Pmin (I) Moisture transport (kg/(ms)) Pmax - Pmin

Supplementary Figure 6: Results of idealised sensitivity precession experiments using high-resolution fully coupled ocean-atmosphere model EC-Earth. Figures were created using the model output and the program Ferret, version 6.82 (version for Mac). Ferret is a tool developed by NOAA, http://www.ferret.noaa.gov/Ferret/. Sea level pressure in hPa for DJF precession maximum (A) and precession minimum (B). Difference between maximum and minimum precession (C). Net precipitation in mm/day for DJF precession maximum (D) and minimum (E). Difference between precession maximum and minimum (F). Moisture transport for DJF precession maximum (G) and precession minimum (H) Difference between precession maximum and minimum (I). Average vector length is 80 kg/(ms), moisture transport is integrated up to 500hPa (approx. 5km).

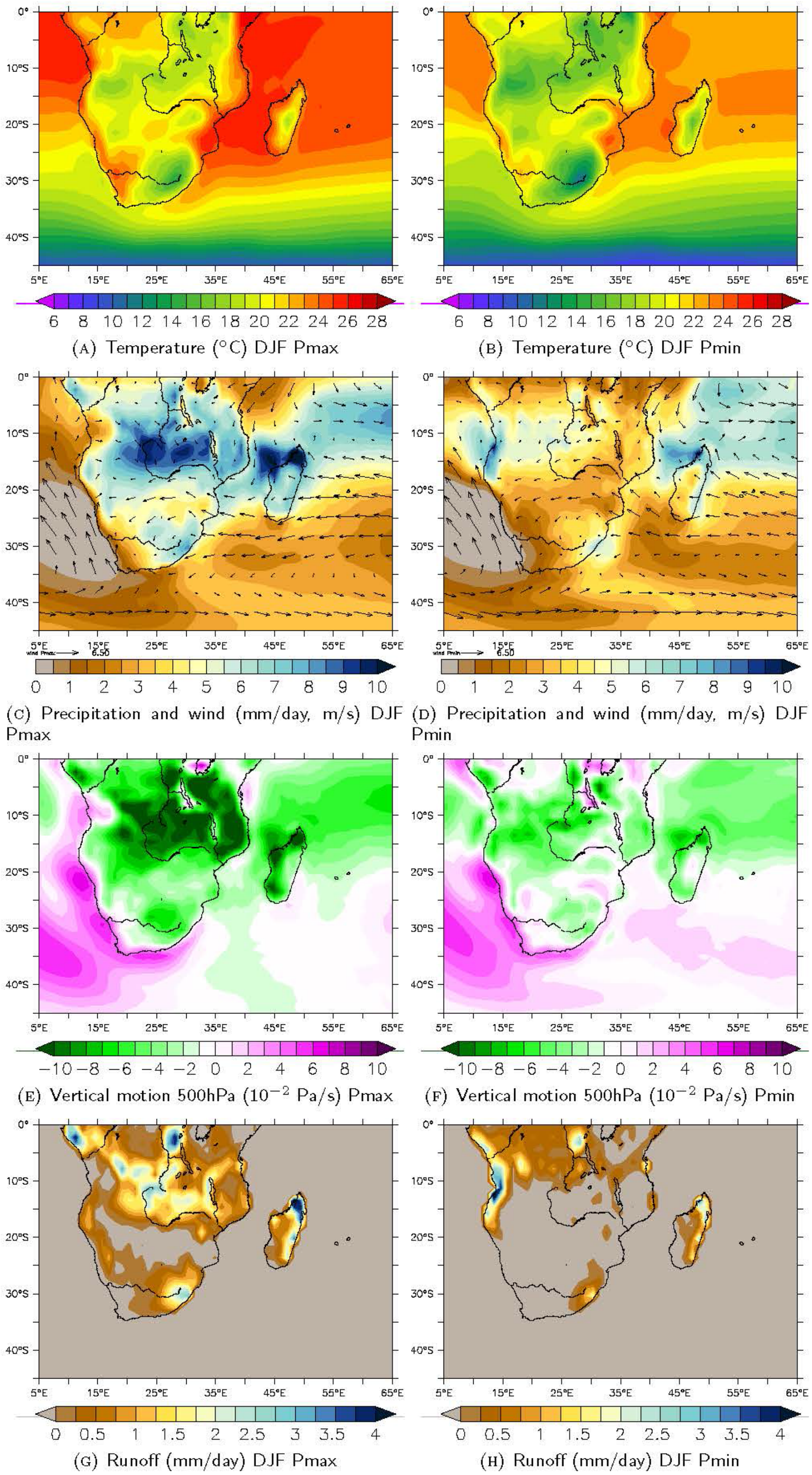

Supplementary Figure 7: Results of idealised sensitivity precession experiments using high-resolution fully coupled ocean-atmosphere model EC-Earth. Figures were created using the model output and the program Ferret, version 6.82 (version for Mac). Ferret is a tool developed by NOAA,

http://www.ferret.noaa.gov/Ferret/. Temperature in Celcius for (December, January, February (DJF)) precession maximum (A) and minimum (B). Precipitation in mm/day for DJF precession maximum (C) and minimum (D) as well as wind (m/s). Vertical motion at 500 hPa (roughly 5km height) is given in 10^{-2} Pa/s for precession maximum (E) and precession minimum (F). Negative values indicate upward motion, positive values indicate downward motion. Surface runoff over land in mm/day for DJF precession maximum (G) and minimum precession (H).

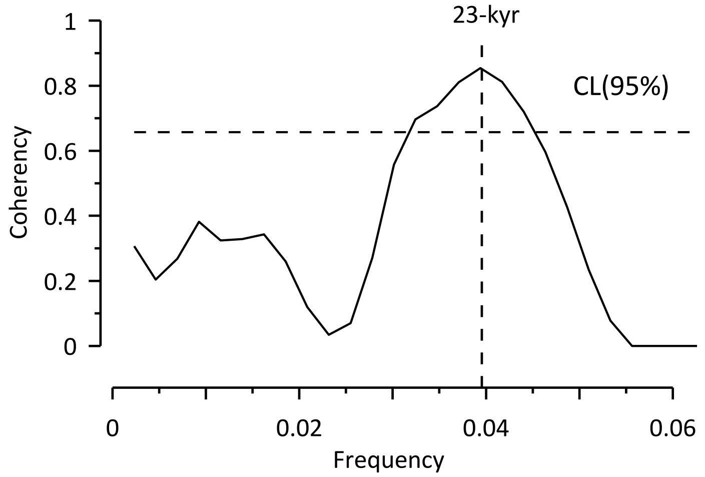

Supplementary Figure 8: Coherence levels from the cross-spectral analysis calculated with SPECTRUM software[6] of the Fe/K record of core CD154 10-06P and Chinese speleothems $\delta^{18}O$ records.

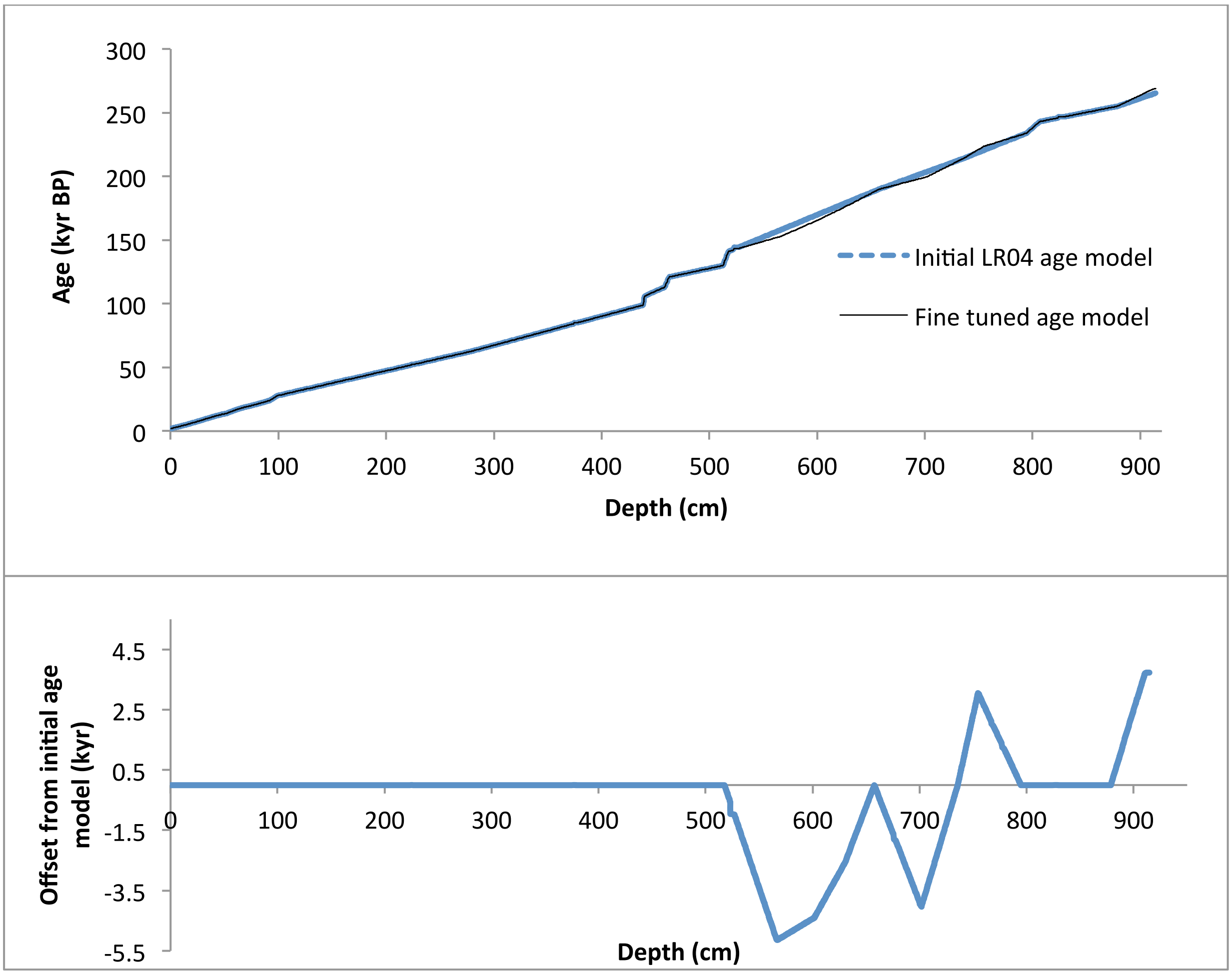

Supplementary Figure 9: Comparison of chronologies for core CD154 10-06P. Upper panel displays age-depth plot for initial LR04 based and fine-tuned age model. Lower panel shows the age offset with respect to the fined- tuned Chinese speleothem $\delta^{18}O$ record based one.

The Chinese speleothem record/ normalisation approach

In general, the $\delta^{18}O$ record from Chinese speleothems has been interpreted to represent the strength of the East Asian Monsoon (EAM) combining the composite effects of precipitation sources that vary with Northern Hemisphere summer insolation at 65 °N[12,13] apparently with a time lag[14, e.g., 15]. It has been shown that the Chinese speleothem $\delta^{18}O$ record lags maximum Northern Hemisphere summer insolation (minimum precession) by ~2.9 ± 0.3 kyr at the precession cycle (Wang et al., 2008). Variations in the $\delta^{18}O$ records in the EAM region are still widely debated as to what they ultimately represent, because, among other reasons, a significant component of EAM precipitation is thought to be derived from the Indian Summer Monsoon domain e.g. [15,16] or reflect annual variations in hydrologic processes and circulation regime over a large part of the Indo-Asian region on orbital timescales[17].

With the aim to locate the timing of events when the EAM responded to abrupt high-latitude forcing the variability of the EAM record needs to be highlighted to this type of forcing, as opposed to the variability attributed to orbital forcing[13]. For this, the insolation component of the EAM has been removed, i.e., 21st of July at 65 °N, a procedure previously proposed by Barker, et al. [18]. We acknowledge that there is currently some discussion about the precise insolation target that should be used[12,15]. The same procedure has been applied to the Fe/K record of core CD154 10-06P by subtracting the local austral summer insolation at 30 °S, as the southeast African records appear to be sensitive to Southern Hemispheres insolation at their specific latitude [19-21]. For both the speleothem $\delta^{18}O$ and the insolation series, the mean value of the series was subtracted from each datum, and divided by the series' standard deviation. Then, the normalised values of insolation were subtracted from the coeval speleothem counterpart. This has been repeated for the Fe/K record of core CD154 10-06P. Transferring the speleo-chronology of core CD 154 10-06P to the record of benthic $\delta^{18}O$ enables an evaluation of the discrepancies between a ^{230}Th-derived chronology and the LR04 isotopic stack which is widely used as tuning target for marine records. The comparison shows that, despite the different tuning approach, a high level of synchronicity between the benthic $\delta^{18}O$ record of core CD154 10-06P and the LR04 record is achieved (Pearson T= 0.919; CL 95% (0.86; 0.95)).

Elemental records

Diagenetic Fe remobilization can, however, occur during redox processes in the sediment[22,23] which will be in form of Fe-Mn oxyhydroxides dispersed as micronodules in the sediments and as coatings on sedimentary particles[24]. Fe-Mn oxides are precipitated directly from seawater, from interstitial water during diagenesis and from oxygenated hydrothermal fluids, and concentrate trace elements greatly[24]. Evidence for diagenetic processes in the deep-sea can be gained by assessing downcore Fe concentrations and their co-variation with Mn as this is also produced during redox processes[22]. Two intervals between 25-30cm and 37-40 cm show elevated XRF Mn counts (Supplementary Figure 10). While, neither interval occurs coevally with elevated Fe concentrations suggesting limited diagenetic overprint, the latter Mn peak occurs directly after a maximum in Fe concentrations centred at ~42 cm core depth. While a diagentic influence on this Fe peak cannot be ruled out it appears of secondary importance to change in supply of the terrigenous components. The down core Mn profile is in strong contrast to the Fe profile, thereby suggesting that the variability in the Fe profile is unlikely to be driven by diagenetic processes

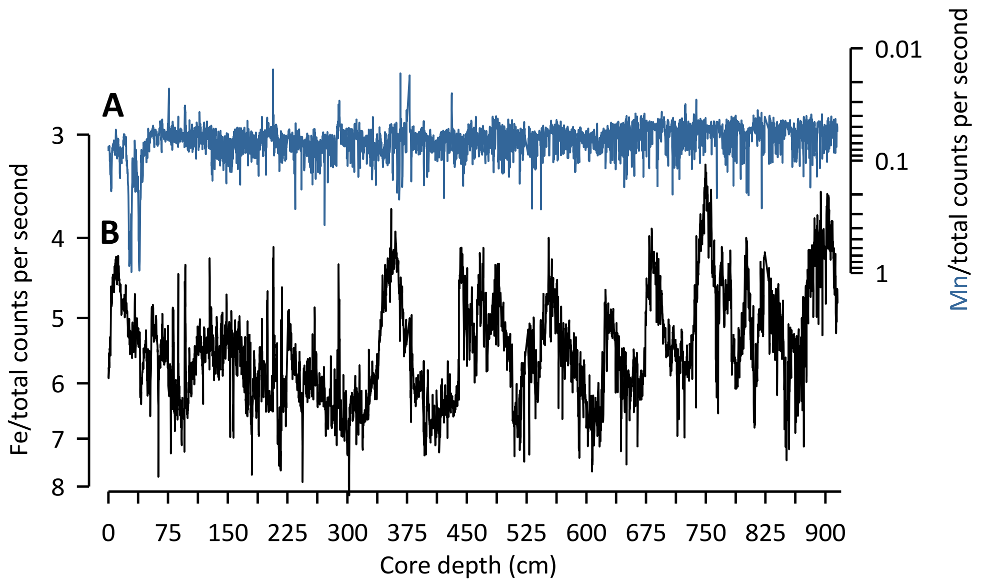

Supplementary Figure 10: Additional XRF scanning profile from marine sediment core CD154 10-06P. (A) Mn normalised (Mn/ total counts per second; blue) (B) Fe normalised (Fe/ total counts per second; black).

Geochemical characteristics of the southeast African river catchments and core location

Variations in Fe/K ratios are interpreted as an indicator for changes between humid and dry conditions in the southeast African hinterland[25,26]. The spatial distributions of Fe/K in marine

core-top sediments reflect the relative input of intensively weathered material from humid regions versus less weathered particles from drier areas[25]. In tropical and subtropical humid regions, intense chemical weathering of bedrocks takes place due to high precipitation and temperatures[22,27]. This leads to the presence of highly weathered soils with a geochemical signature that is rich in Fe[27] and which is transferred to marine sediments by fluvial input. Potassium in marine sediments is mostly associated with potassium feldspar ($K[AlSi_3O_8]$),[28] or illite ($(K,H_3O)Al_2[(OH)_2Si_3AlO_{10}]$),[29], which are both characteristic of drier regions with low chemical weathering rates and in which physical weathering is dominant[28]. Enhanced chemical weathering and iron oxide mobilisation, as well as soil erosion, on land requires frequent rainfall over the continent and so are indicative of more humid conditions in the hinterland climate. Elevated chemical weathering rates, due to increased precipitation, result in highly-weathered soils typical for tropic and subtropical environments. As clay minerals are intensely weathered, the structure of silicate clays change as they lose silica and the remaining soil is enriched in iron oxides, such as Goethite ($FeO(OH)$) or Hematite (Fe_2O_3),[30] which is ultimately recorded as high Fe/K values in the sediment. Conversely, drier conditions in the hinterland favour reduced chemical weathering, resulting in low Fe/K ratios in marine sediments[25]. Therefore the Fe/K ratio of surface sediments should be appropriate for reconstructing African climatic zones[25] but importantly does not allow any quantitative estimate of terrigenous material supplied to the ocean via the river system. However, in comparison to the use of Fe/Ca ratios this method is independent of possible variations in biogenic carbonate input or post-depositional calcium carbonate dissolution. Owing to the prevailing easterly winds, aeolian transport of terrigenous material from subtropical southern Africa towards the southwest Indian Ocean is considered negligible[31]. Previous studies have confirmed that marine sediment cores situated along the southeast African continental margin are sensitive to local river discharge and can be used to reconstruct past variations in the continental climate regime using variations in the bulk elemental concentrations such as Fe/K[20,32,33].

BIT index

Branched Glycerol dialkyl glycerol tetraether (GDGT) seems to occur predominantly in the terrestrial environment (i.e., soils, peats, lakes and rivers), while crenarchaeol is thought to be the dominant GDGT in the marine environment. Consequently, index ratios are close to 1 in soils and peats, whereas values close to 0 are found in open marine sediments[34]. Later studies showed that the index was not as much a measure of total terrestrial organic matter (OM) but

rather a measure of only soil OM, since branched GDGTs were ubiquitously present in soil[35] and the index did not correlate well with other terrestrial proxies ($\delta_{13}C_{org}$) in sediments[36]. The BIT index has therefore been frequently used to trace the input of soil OM via fluvial transport into marine environments[37-39].

Samples were freeze-dried and homogenized with a mortar and pestle. The homogenized material was then extracted using an accelerated solvent extractor with dichloromethane (DCM):methanol 9:1 (*v/v*) and a pressure of 1000 psi in 3 extraction cycles. The total lipid extract was separated over an Al_2O_3 column into a polar, ketone and polar fraction using hexane:DCM 9:1, hexane:DCM 1:1 and DCM:methanol 1:1, respectively. The polar fractions were redissolved in hexane/isopropanol (99:1) to a concentration of 2 mg/ml and filtered over a 0.45 PTFE filter and analyzed for the GDGT lipid based TEX_{86} and BIT index using high performance liquid chromatography mass spectrometry (HPLC/MS). Analyses for GDGTs were performed as described by Schouten et al. [40]. In summary, an Agilent 1100 series HPLC/MS equipped with an auto-injector and Agilent Chemstation chromatography manager software was used. Separation was achieved on an Alltech Prevail Cyano column (2.1mm×150mm, 3 µm), maintained at 30 °C. GDGTs were eluted with 99 % hexane and 1 % propanol for 5 min, followed by a linear gradient to 1.8 % propanol in 45 min. Flow rate was 0.2 mL min^{-1} by back-flushing hexane/propanol (90:10, *v/v*) at 0.2 mL min^{-1} for 10 min. Detection was achieved using atmospheric pressure positive ion chemical ionization mass spectrometry (APCI-MS) of the eluent. Conditions for the Agilent 1100 APCI-MS 5 were as follows: nebulizer pressure of 60 psi, vaporizer temperature of 400 °C, drying gas (N2) flow of 6 $Lmin^{-1}$ and temperature 200 °C, capillary voltage of −3kV and a corona of 5 µA (~3.2 kV). GDGTs were detected by Single Ion Monitoring (SIM) of their $[M+H]^+$ ions (dwell time = 234ms), [40] and quantified by integration of the peak areas. BIT index was calculated as described by [34]. The analytical error for BIT index is ± 0.01. Concentrations of GDGTs were calculated by reference to a C_{46} GDGT internal standard [41].

Supplementary references

1 Lisiecki, L. E. & Raymo, M. E. A Pliocene-Pleistocene stack of 57 globally distributed benthic $d^{18}O$ records. *Paleoceanography* **20**, PA1003, doi:10.1029/2004pa001071 (2005).

2 Parnell, A. C., Haslett, J., Allen, J. R. M., Buck, C. E. & Huntley, B. A flexible approach to assessing synchroneity of past events using Bayesian reconstructions of sedimentation history. *Quaternary Science Reviews* **27**, 1872-1885, doi:10.1016/j.quascirev.2008.07.009 (2008).

3 Mudelsee, M. Estimating Pearson's Correlation Coefficient with Bootstrap Confidence Interval from Serially Dependent Time Series. *Mathematical Geology* **35**, 651-665, doi:10.1023/B:MATG.0000002982.52104.02 (2003).

4 Schulz, M. & Mudelsee, M. REDFIT: estimating red-noise spectra directly from unevenly spaced paleoclimatic time series. *Computers & Geosciences* **28**, 421-426, doi:10.1016/S0098-3004(01)00044-9 (2002).

5 Mann, M. E. a. & Lees, J. M. Robust estimation of background noise and signal detection in climatic time series. *Climatic Change* **33**, 409-445 (1996).

6 Schulz, M. & Stattegger, K. Spectrum: spectral analysis of unevenly spaced paleoclimatic time series. *Computers & Geosciences* **23**, 929-945, doi:10.1016/S0098-3004(97)00087-3 (1997).

7 Paillard, D., Labeyrie, L. & Yiou, P. Macintosh Program performs time-series analysis. *Eos, Transactions American Geophysical Union* **77**, 379-379, doi:10.1029/96EO00259 (1996).

8 Berger, A. L. Support for the astronomical theory of climatic change. *Nature* **269**, 44-45 (1977).

9 Milankovitch, M. Mathematische Klimalehre und Astronomische Theorie der Klimaschwankungen. *Handbuch der Klimalogie, Borntrager Berlin* **Band 1** (1930).

10 Berger, A. Long-term variations of daily insolation and Quaternary climatic changes. *J. Atmos. Sci.* **35**, 2362-2367 (1978).

11 Hammer, Ø., Harper, D.A.T., and P. D. Ryan, . PAST: Paleontological Statistics Software Package for Education and Data Analysis. *Palaeontologia Electronica* **4**, 9 (2001).

12 Ziegler, M., Tuenter, E. & Lourens, L. J. The precession phase of the boreal summer monsoon as viewed from the eastern Mediterranean (ODP Site 968). *Quaternary Science Reviews* **29**, 1481-1490, doi:10.1016/j.quascirev.2010.03.011 (2010).

13 Wang, Y. *et al.* Millennial- and orbital-scale changes in the East Asian monsoon over the past 224,000 years. *Nature* **451**, 1090-1093, doi:10.1038/nature06692 (2008).

14 Cheng, H., Sinha, A., Wang, X., Cruz, F. & Edwards, R. L. The Global Paleomonsoon as seen through speleothem records from Asia and the Americas. *Climate Dynamics* **39**, 1045-1062, doi:10.1007/s00382-012-1363-7 (2012).

15 Clemens, S. C., Prell, W. L. & Sun, Y. Orbital-scale timing and mechanisms driving Late Pleistocene Indo-Asian summer monsoons: Reinterpreting cave speleothem $\delta^{18}O$. *Paleoceanography* **25**, PA4207, doi:10.1029/2010PA001926 (2010).

16 Pausata, F. S. R., Battisti, D. S., Nisancioglu, K. H. & Bitz, C. M. Chinese stalagmite $d^{18}O$ controlled by changes in the Indian monsoon during a simulated Heinrich event. *Nature Geosci* **4**, 474-480, doi:10.1038/ngeo1169 (2011).

17 Caley, T., Roche, D. M. & Renssen, H. Orbital Asian summer monsoon dynamics revealed using an isotope-enabled global climate model. *Nat Commun* **5**, doi:10.1038/ncomms6371 (2014).

18 Barker, S. *et al.* 800,000 Years of Abrupt Climate Variability. *Science* **334**, 347-351, doi:10.1126/science.1203580 (2011).

19 Partridge, T. C., de Menocal, P. B., Lorentz, S. A., Paiker, M. J. & Vogel, J. C. Orbital forcing of climate over South Africa: A 200,000-year rainfall record from the pretoria saltpan. *Quaternary Science Reviews* **16**, 1125-1133, doi:10.1016/S0277-3791(97)00005-X (1997).

20 Schefuß, E., Kuhlmann, H., Mollenhauer, G., Prange, M. & Patzold, J. Forcing of wet phases in southeast Africa over the past 17,000 years. *Nature* **480**, 509-512, doi:10.1038/nature10685 (2011).

21 Trauth, M. H., Deino, A. L., Bergner, A. G. N. & Strecker, M. R. East African climate change and orbital forcing during the last 175 kyr BP. *Earth and Planetary Science Letters* **206**, 297-313, doi:10.1016/S0012-821X(02)01105-6 (2003).

22 Middelburg, J. J., van der Weijden, C. H. & Woittiez, J. R. W. Chemical processes affecting the mobility of major, minor and trace elements during weathering of granitic rocks. *Chemical Geology* **68**, 253-273, doi:10.1016/0009-2541(88)90025-3 (1988).

23 Zwolsman, J. J. G. & van Eck, G. T. M. Geochemistry of major elements and trace metals in suspended matter of the Scheldt estuary, southwest Netherlands. *Marine Chemistry* **66**, 91-111, doi:10.1016/S0304-4203(99)00026-2 (1999).

24 Libes, S. M. Introduction to Marine Biogeochemistry. *Elsevier,* **2nd edition** (2009).

25 Govin, A. *et al.* Distribution of major elements in Atlantic surface sediments (36°N–49°S): Imprint of terrigenous input and continental weathering. *Geochemistry, Geophysics, Geosystems* **13**, Q01013, doi:10.1029/2011GC003785 (2012).

26 Mulitza, S. *et al.* Sahel megadroughts triggered by glacial slowdowns of Atlantic meridional overturning. *Paleoceanography* **23**, PA4206, doi:10.1029/2008PA001637 (2008).

27 Driessen, P., Deckers, J., O. Spaargaren & Nachtergaele, F. Lecture Notes on the Major Soils of the World. *Food and agriculture organization of the United Nations Rome* (2001).

28 Zabel, M. *et al.* Late Quaternary Climate Changes in Central Africa as Inferred from Terrigenous Input to the Niger Fan. *Quaternary Research* **56**, 207-217, doi:10.1006/qres.2001.2261 (2001).

29 Yarincik, K. M., Murray, R. W. & Peterson, L. C. Climatically sensitive eolian and hemipelagic deposition in the Cariaco Basin, Venezuela, over the past 578,000 years: Results from Al/Ti and K/Al. *Paleoceanography* **15**, 210-228, doi:10.1029/1999PA900048 (2000).

30 Schaetzl, R. J. & and Anderson, S. Soils: Genesis and Morphology. *Cambridge University Press, Cambridge* (2005).

31 Nicholson, S. E. The nature of rainfall variability over Africa on time scales of decades to millenia. *Global and Planetary Change* **26**, 137-158, doi:10.1016/S0921-8181(00)00040-0 (2000).

32 van der Lubbe, J. J. L. *et al.* Sedimentation patterns off the Zambezi River over the last 20,000 years. *Marine Geology* **355**, 189-201, doi:10.1016/j.margeo.2014.05.012 (2014).

33 Ziegler, M. *et al.* Development of Middle Stone Age innovation linked to rapid climate change. *Nat Commun* **4**, 1905, doi:10.1038/ncomms2897 (2013).

34 Hopmans, E. C. *et al.* A novel proxy for terrestrial organic matter in sediments based on branched and isoprenoid tetraether lipids. *Earth and Planetary Science Letters* **224**, 107-116, doi:10.1016/j.epsl.2004.05.012 (2004).

35 Weijers, J. W. H. *et al.* Membrane lipids of mesophilic anaerobic bacteria thriving in peats have typical archaeal traits. *Environmental Microbiology* **8**, 648-657, doi:10.1111/j.1462-2920.2005.00941.x (2006).

36 Huguet, C., Smittenberg, R. H., Boer, W., Sinninghe Damsté, J. S. & Schouten, S. Twentieth century proxy records of temperature and soil organic matter input in the Drammensfjord, southern Norway. *Organic Geochemistry* **38**, 1838-1849, doi:10.1016/j.orggeochem.2007.06.015 (2007).

37 Kim, J.-H. *et al.* Origin and distribution of terrestrial organic matter in the NW Mediterranean (Gulf of Lions): Exploring the newly developed BIT index. *Geochemistry, Geophysics, Geosystems* **7**, Q11017, doi:10.1029/2006GC001306 (2006).

38 Kim, J.-H. *et al.* Tracing soil organic carbon in the lower Amazon River and its tributaries using GDGT distributions and bulk organic matter properties. *Geochimica et Cosmochimica Acta* **90**, 163-180, doi:10.1016/j.gca.2012.05.014 (2012).

39 Herfort, L. *et al.* Characterization of transport and deposition of terrestrial organic matter in the southern North Sea using the BIT index. *Limnol. Oceanogr.* **51**, 2196-2205, doi:10.4319/lo.2006.51.5.2196 (2007).

40 Schouten, S., Huguet, C., Hopmans, E. C., Kienhuis, M. V. & Damsté, J. S. Analytical methodology for TEX_{86} paleothermometry by high-performance liquid chromatography/atmospheric pressure chemical ionization-mass spectrometry. *Analytical chemistry* **79**, 2940-2944, doi:10.1021/ac062339v (2007).

41 Huguet, C. *et al.* An improved method to determine the absolute abundance of glycerol dibiphytanyl glycerol tetraether lipids. *Organic Geochemistry* **37**, 1036-1041, doi:10.1016/j.orggeochem.2006.05.008 (2006).
